# Acute Myocardial Infarction Complicating Active Ulcerative Colitis: A Case Report

**DOI:** 10.1155/2011/876896

**Published:** 2011-08-08

**Authors:** Eva D. Papadimitraki, Mubarak Ahamed, Nicholas H. Bunce

**Affiliations:** Department of Cardiology, St George's Hospital, Blackshaw Road, London SW17 0QT, UK

## Abstract

Ulcerative colitis (UC) is a chronic inflammatory disease that predominantly affects the gastrointestinal (GI) tract but can involve extraintestinal organs including musculoskeletal system and skin. The most frequent cardiac manifestations of UC are pericarditis and myocarditis. Patients display an increased risk for venous thromboembolic complications and mesenteric ischemia, but the association with ischemic heart disease and myocardial infarction is uncertain. We present the case of a 27-year-old man with anti-PRIII ANCA-positive ulcerative colitis and increased factor VIII activity who presented with an acute myocardial infarction. We discuss possible causative links between these clinical entities and demonstrate the role of cardiac magnetic resonance (CMR) in patients with underlying inflammatory conditions who present with chest pain and evidence of myocardial damage.

## 1. Case Presentation

A 27-year-old Afro-Caribbean male with ulcerative colitis (UC) was admitted with a week's history of intermittent chest pain.

The chest pain was stabbing in nature, with radiation to the neck and left arm, and had increased in intensity 24 hours prior to admission. There was no pleuritic component or accompanying dyspnoea. 

The patient had suffered a recent flare of his UC with bloody diarrhea and malaise. He had been switched from steroids to azathioprine (AZA) 2.5 weeks prior to admission following a normal thiopurine S-methyltransferase (TPMT) assay. He was also receiving mesalazine.

There were no smoking, hypertension, dyslipidemia, diabetes, or family history of coronary artery disease, and the patient denied any use of illicit drugs. His sister suffered from an “undifferentiated polyarthritis” under no treatment, and there was a positive family history for early pregnancy loss.

On admission, he was afebrile, with a blood pressure of 110/70 and heart rate of 80. He was breathing at a rate of 18 breaths per minute, and his oxygen saturation was 97% in room air. Heart sounds were normal. Jugular venous pressure was not raised, and lungs were clear. His abdomen was soft and diffusely tender to palpation. There was evidence of sacroiliitis with reduced abduction of right hip, but chest expansion, lumbar spinal movements, and peripheral joints were intact. There were no rashes. 

**Figure 1:**
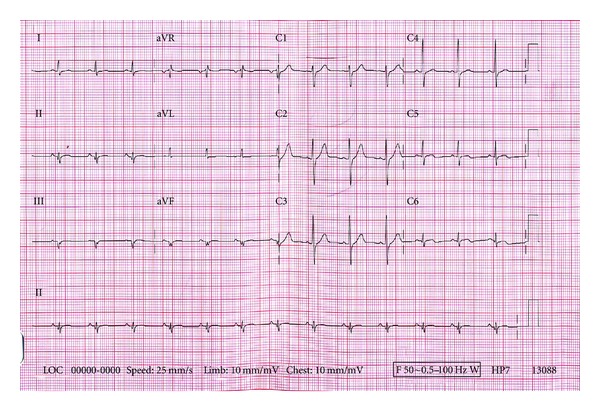
Electrocardiogram on admission reveals pathological q waves and nonspecific ST-T wave changes of unknown age in the inferior leads (II, III, and aVF) and early precordial R/S transition suggesting concomitant posterior wall participation.

Electrocardiogram (ECG) on admission demonstrated sinus rhythm, 80 bpm, with normal axis and pathological q waves in the inferior leads and tall R waves in leads V1–V4 (Figure 1).

Troponin I was elevated on admission (>50.00 mg/mL). Blood analysis revealed a marked inflammatory response with anemia (Hb: 11.4 g/dL, reference range: 13.0–18.0 g/dL), increased platelets (465 × 10^9^/L, reference range: 150–450 × 10^9^/L), low albumin (31 g/L, reference range: 35–48 g/L), and marginally increased peripheral eosinophils (1.1 × 10^9^/L, reference range 0.1–0.8 × 10^9^/L). Blood cultures were negative, and serology was negative for active or recent viral (Echo, Coxsackie, EBV, CMV) or Streptococcal infection. Rheumatoid factor (RF) was positive (31 iu/mL, reference range: 0–20 iu/mL), but antinuclear antibodies (ANA), mitochondrial, smooth muscle, and liver microsomal antibodies were all negative, and complement fractions (C3 and C4) were not decreased. Antiproteinase III (anti-PR-III) antibodies were positive in high titers (10.2 u/mL, reference range 0–2 u/mL), but perinuclear antineutrophil cytoplasmic antibodies (P-ANCA) were negative. Chest X-ray was normal. Urine dipstick was negative for blood or protein. 

Flexible sigmoidoscopy showed diffuse mildly active colitis, and biopsies obtained confirmed the presence of chronic active distal colitis with no ulceration, granuloma formation, dysplasia, or malignancy. 

Transthoracic echocardiography on admission (TEE) showed an overall preserved systolic function with inferolateral and anterolateral wall hypokinesia. There was mild mitral regurgitation. There was no pericardial effusion.

Differential diagnosis included myocarditis (inflammatory-bowel-disease-(IBD-) related giant cell myocarditis, drug-induced hypersensitivity myocarditis, or acute lymphocytic myocarditis) or late presentation myocardial infarction (MI). 

In view of his young age, the patient was referred for cardiovascular magnetic resonance (CMR), which was performed on a 1.5 T GE SIGNA system with 16-element chest coil. Function was assessed using steady-state free precession (SSFP) in short axis slices covering the ventricles and in 4-chamber, 2-chamber, and left ventricular outflow tract (LVOT) orientations. Multiple Double Inversion (IR) Fast-Spin ECHO black-blood images were acquired. Late gadolinium enhancement (LGE) images were obtained following gadolinium 0.1 mmol/kg administration (Dotarem, Guerbet, CEDEX) after 10–20 min in short axis and 4 chamber orientations. Inversion time was adjusted to null the signal from normal LV myocardium.

**Figure 2:**
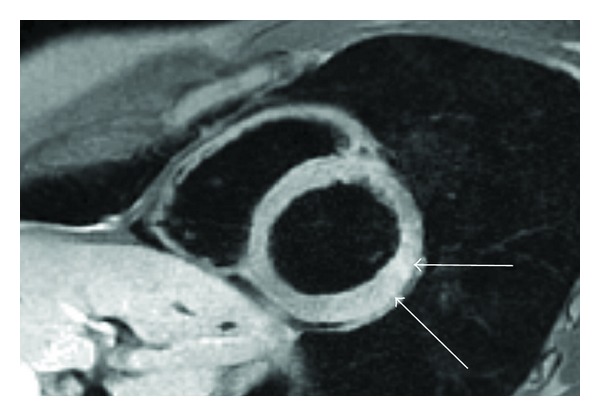
Double inversion recovery FSE. Short axis slice demonstrates increased signal intensity (white arrows) in the basal-mid lateral wall consistent with myocardial oedema.

**Figure 3:**
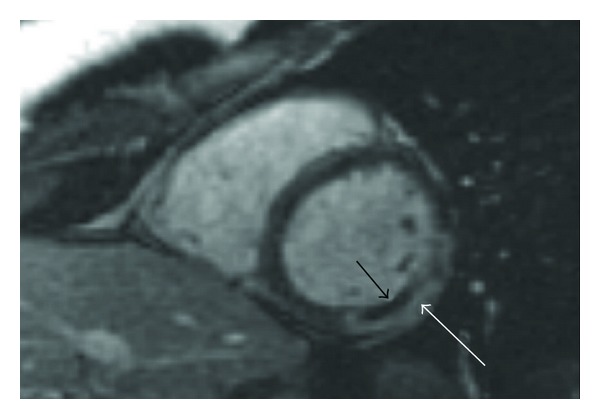
Late gadolinium enhancement. Short axis slice. Following gadolinium, there is transmural delayed enhancement (white arrow) in the basal-mid lateral wall with a central core of noenhancement (black arrowhead) consistent with a recent myocardial infarction with a zone of no-reflow.

The CMR study showed mildly impaired left systolic function with left ventricular ejection fraction (EF) of 53% and hypokinesia of the basal-mid inferolateral wall (accompanying MOV file, see MOV file in Supplementary Material available online at doi: 10.1155/2011/876896). Left ventricular volumes were preserved. On Double IR FSE images, myocardial signal intensity appeared increased in the hypokinetic segments (Figure 2) and there was 100% transmural delayed enhancement with a central zone of hypoenhancement in the LGE study (Figure 3). Left atrium (LA), right atrium (RA), and right ventricle (RV) appeared normal in size and function. Mitral and aortic valves were intact, and there was no pericardial effusion.

**Figure 4:**
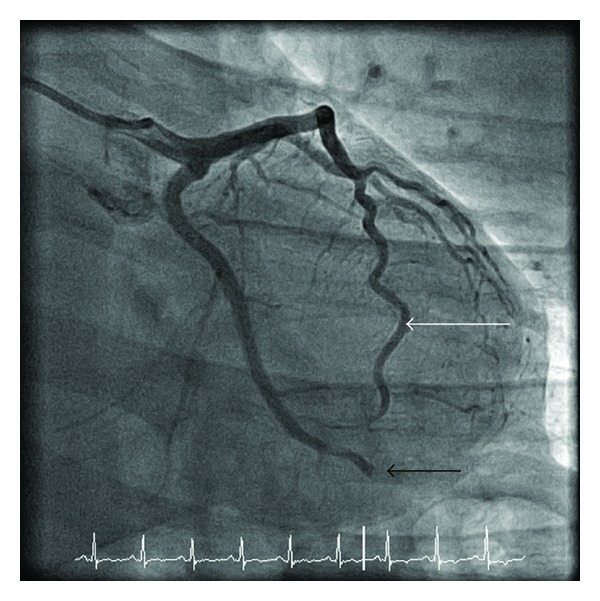
Diagnostic coronary angiography demonstrated an unobstructed left anterior descending coronary artery (white arrow) and distal occlusion of a nondominant left circumflex coronary artery (black arrowhead). The dominant right coronary artery (not shown) was normal.

The CMR appearances were consistent with a recent myocardial infarction, and, on this basis, cardiac catheterization was performed. This demonstrated distal occlusion of a nondominant left circumflex coronary artery with otherwise unobstructed coronary arteries (Figure 4). Because of the completed infarct, percutaneous coronary intervention (PCI) was not performed. 

The patient was initially started on aspirin, bisoprolol, ramipril, simvastatin, and low-molecular-weight heparin. A thrombophilia screen including measurement of protein C, protein S, antithrombin III levels, activated protein C (APC) resistance, antiphospholipid antibodies and lupus anticoagulant, factor VIII activity and genetic analysis for Factor Leiden, prothrombin gene mutation, and methylenetetrahydrofolate (MTHFR) polymorphism was performed. Apart from an increased factor VIII clotting activity (159 iu/dL, reference range 50–150 iu/dL), these tests were within normal limits. Bubble contrast transthoracic echocardiogram was negative for patent foramen ovale.

The patient was subsequently started on warfarin, and the dose of mesalazine was increased. His gastrointestinal symptoms significantly improved, and he was then discharged after experiencing no further chest pain. He remains well and is currently being assessed as a candidate for disease modifying antirheumatic drugs (DMARDs) or alternatively antitumor necrosis alpha (anti-TNF-*α*) agents for his combined inflammatory conditions. 

The clinical diagnosis was late presentation MI associated with intra-arterial thrombosis, mildly active UC with concomitant enteropathic spondyloarthropathy, possible medium vessel vasculitis and secondary elevated factor VIII activity. 

## 2. Discussion

 Ulcerative colitis is a chronic relapsing and remitting inflammatory disorder of the colonic mucosa that is often associated with extraintestinal manifestations from the skin, liver, eyes, kidneys, and joints. Myocarditis and myopericarditis are the most common cardiovascular manifestations of UC, although their overall prevalence in patients with IBD remains largely unknown. Heart amyloidosis, endomyocardial fibrosis, and dilated cardiomyopathy have also been described [1]. A possible association between ischemic heart disease (IHD) and IBD was first suggested by a Finish epidemiological study that found a significantly increased prevalence of coronary artery disease among men and women with IBD compared to age- and sex-matched healthy controls [2]. Although there have been scarce reports of MI in patients with UC in the literature [3, 4], a recently published retrospective study assessing the risk of arterial thrombosis in 17,487 IBD patients and 69,948 controls found no overall increased prevalence of MI in IBD patients. However, subgroup analysis of populations under study showed a higher risk of MI among women over 40 with IBD (similar risks for UC and CD, hazard ratio (HR), 1.6, *P* = 0.003). Two of the inherent drawbacks of the above-mentioned study have been that it failed to control for traditional risk factors for CHD and that it provided no information as to whether MI was prominently atherogenic or not, and thus its findings need to be interpreted cautiously [5]. In conclusion, the evidence for a clear association between UC/IBD and MI remains rather unclear at present.

 Our patient exhibited an intense inflammatory burden, as evidenced by the increased inflammatory markers, the presence of PR-III ANCA and RF, and the increased factor VIII activity. It is conceivable that inflammation-induced activation of the coagulation cascade and cytokine (e.g., interleukin-6 (IL-6) and TNF-*α*) mediated tissue factor activation may additively promote a precoagulant state resulting in vascular events in patients with IBD flares [6, 7]. Ulcerative colitis has been traditionally linked to the presence of perinuclear (P) rather than proteinase III (PR-III) ANCA, and although the latter have been associated with Wegener's granulomatosis and recently large vessel vasculitides, their pathogenicity remains unclear. It has been however shown that they are in vitro capable of enhancing endothelial/neutrophil interactions, which implies their role in endothelial activation and vascular inflammation [8]. Factor VIII normally functions as a cofactor for Factor IX during the fluid phase of the coagulation system and is associated with perturbed endothelial function and platelet aggregation [9]. Plasma concentrations above 150 IU/dL—either genetically predetermined or in the context of an acute phase response—have been shown to increase the risk of thrombosis by a factor of 6, and patients with incremented factor VIII levels may exhibit shorted survival after stroke or MI [10]. 

A variety of integrating signals employing acute phase proteins, autoantibodies, and coagulation factors may act in concert to induce an inflammatory/procoagulant state accounting for the thrombotic burden in patients with underlying inflammatory conditions such as IBD, even in the absence of traditional cardiovascular risk factors. Extrapolating data from rheumatoid arthritis, it could be postulated that aggressive anti-inflammatory treatment could reduce the thrombotic burden in patients with IBD, but this remains to be shown [11]. Tumor necrosis factor (TNF)-*α* blocking agents may represent a reasonable therapeutic option against multiorgan inflammation (joints, intestine, and blood vessels) in the absence of stage III/IV heart failure. Regarding the optimal duration of anticoagulation in view of the increased plasma VIII activity, this remains largely empirical and individualized among cases; an initial schedule for 6 months on warfarin with further reassessment of thrombotic risk at the time is generally acceptable, unless otherwise indicated. 

In addition, previous studies have identified a role for CMR in the investigation of patients presenting with chest pain and raised biomarkers with unobstructed coronary arteries [12]. However, in young patients with chest pain and atypical features, it may be appropriate to perform CMR as the initial investigation to identify cases with nonischaemic conditions who might avoid the risks of unnecessary cardiac catheterization or percutaneous coronary intervention.

## 3. Conclusions

We present a case of myocardial infarction in a patient with UC and enteropathic arthritis who showed evidence of vascular inflammation (anti-PRIII antibodies) and activation of the coagulation cascade (increased factor VIII activity). Myopericarditis is the most prevalent cardiac manifestation in young patients with underlying IBD. However, they may also display an increased thrombotic/inflammatory burden resulting in atherogenic or nonatherogenic MIs. A high index of clinical suspicion and a low threshold for early imaging in such patients presenting with chest pain and evidence of myocardial damage are thus recommended.

##  Consent

Written informed consent was obtained from the patient for publication of this case report and accompanying images. A copy of the written consent is available for review by the Editor-In-Chief of this journal.

##  Conflict of Interests

The authors declare that they have no competing interests.

##  Authors' Contributions

E. D. Papadimitraki was involved in collecting the patient's clinical data and drafting the paper. M. Ahamed participated in data collection. N. H. Bunce performed the CMR study and was involved in image data collection and paper revision. All authors have seen and approved the final version of the paper.

## Supplementary Material

Steady-state free precession (SSFP) cine image. Short axis slice demonstrates hypokinesia of the basal-mid inferolateral wall.
